# Divergent Mechanisms Linking Parental Psychological Control to Perfectionism Among Adolescents: The Longitudinal Role of Need Frustration

**DOI:** 10.3390/bs16071145

**Published:** 2026-07-08

**Authors:** Binxu Wang, Yan Liu

**Affiliations:** Institute of Developmental Psychology, Faculty of Psychology, Beijing Normal University, Beijing 100875, China; binxu.wang@foxmail.com

**Keywords:** parental psychological control, basic need frustration, perfectionism, adolescents, self-determination theory

## Abstract

Parental psychological control is theoretically posited as a vital antecedent of perfectionism, yet empirical research connecting them remains limited, with the underlying mechanisms being particularly underexplored. Specifically, it remains unclear whether parental psychological control is associated with the maladaptive (evaluative concerns) and adaptive (personal striving) facets of perfectionism through convergent or divergent pathways. Three-wave longitudinal design (T1–T3) with six-month intervals was conducted with 1432 adolescents (*M*_age_ = 14.60, *SD* = 1.66, 54% girls) to investigate the direct and indirect effects. Data were analyzed using structural equation modeling, complemented by a random-intercept mediation model to disentangle trait versus state-level effects. Results revealed a distinct pathway linking parental psychological control to perfectionism dimensions via need frustration that is linked to both higher maladaptive and lower adaptive perfectionism. While T1 parental psychological control predicted frustration of all three basic needs in T2, the downstream pathways diverged. Relatedness and competence frustration mediated the link to increased T3 evaluative concerns, whereas autonomy frustration mediated the link to decreased T3 personal striving. Supplementary analysis using a random-intercept mediation model indicated that the overall mediation process was primarily influenced by stable trait-level differences rather than within-person deviations. Findings highlight that parental psychological control differentially predicted perfectionism facets through specific need frustration, with the overall indirect process reflecting primarily a stable, trait-level mechanism.

## 1. Introduction

Over the past three decades, perfectionism has witnessed a substantial generational increase globally (Curran & Hill, 2019). Far from being a unidimensional trait, contemporary research identifies perfectionism as a multidimensional construct, primarily comprising maladaptive and adaptive components (Bieling et al., 2004). While perfectionism can be adaptive, substantial research regards perfectionism as a pervasive, transdiagnostic risk factor that critically predicts adolescent social adaptation, problem behaviors, academic trajectories, and psychological well-being (Curran & Hill, 2022).

The rising prevalence of perfectionism among contemporary youth is widely understood as the product of a complex interplay between personality (Kim et al., 2015), environmental (Sand et al., 2021), and cultural factors (Curran & Hill, 2022). Crucially, while these multifaceted factors set the stage, they are filtered and operationalized through the family system. Within this context, parental psychological control (PPC) is associated with how adolescents internalize these high external standards and has emerged as a critical antecedent of maladaptive adolescent development (Kunz & Grych, 2013). By directly thwarting children’s psychological needs, PPC has been gradually identified as a key environmental predictor of maladaptive perfectionism.

However, while the link between PPC and perfectionism is documented, the precise mechanisms through which PPC translates into specific perfectionistic facets remain unclear. Basic psychological needs theory within Self-Determination Theory (Ryan & Deci, 2017) posits that controlling parenting frustrates the essential basic psychological needs which serve as a primary precursor to psychopathology. Thus, we suggest that perfectionism manifests not merely as passive compliance with parental control, but as a form of introjected regulation to underlying need frustration.

Accordingly, the present study proposes a specific frustrated needs framework to dissect this process. Utilizing a three-wave longitudinal design, we aim to clarify which specific type of need frustration mediates the influence of PPC on perfectionism and to investigate these effects by disentangling stable between-person differences from within-person fluctuations. This approach will illuminate the extent to which frustrated needs mediate the links between PPC and distinct perfectionism components, and whether need frustration serves as a long-term mechanism which PPC shapes the perfectionistic personality structure.

## 2. Parental Psychological Control and Perfectionism Among Adolescents

Perfectionism is broadly defined as a multidimensional personality trait characterized by the setting of excessively high performance standards accompanied by overly critical self-evaluations (Curran & Hill, 2019). To capture the nuanced impact of perfectionism on adolescent development, contemporary research has moved beyond early multifaceted models (e.g., Hewitt & Flett, 1991) toward a parsimonious bi-dimensional framework (Bieling et al., 2004). This framework distinguishes between evaluative concerns (maladaptive), marked by fear of mistakes and external validation, and personal striving, characterized by high standards and mastery goals. Additionally, empirical evidence supports the adaptive nature of striving, which demonstrates that high standards yield psychological benefits when unaccompanied by a debilitating discrepancy between performance and expectations (Slaney et al., 2001). This conceptual distinction is vital during adolescence, a critical developmental window where these dimensions drive divergent trajectories. While the adaptive facet of perfectionism can foster positive social adaptation (Kim et al., 2015), the maladaptive facet exacerbates internalizing symptoms and suicide risk (Smith et al., 2022). Thus, the present study adopts this consolidated framework to elucidate how parental factors uniquely shape these distinct, yet consequential, facets of perfectionism.

PPC refers to an intrusive parenting style involving guilt induction and love withdrawal, and the invalidation of feelings (Soenens et al., 2005); it targets adolescents’ self-worth and severely thwarts basic psychological needs (Soenens & Vansteenkiste, 2010). When parental affection is perceived as conditional, adolescents are compelled to introject high parental expectations to maintain relatedness. Aligning with the social expectation model, when parental affection is perceived as conditional, adolescents learn that worthiness is contingent upon meeting external achievements (Kopala-Sibley & Zuroff, 2014). Hence, they are compelled to internalize these rigid standards to maintain parental relatedness and secure approval. In this context, perfectionism, specifically maladaptive facet, emerges as a defensive strategy to avoid parental criticism (Gong et al., 2016). Existing research has consistently supported the positive association between PPC and maladaptive perfectionism. For instance, Soenens et al. (2005) found that PPC remained the most robust and unique predictor of maladaptive perfectionism even after controlling parental maladaptive perfectionism, responsiveness, and behavioral control. Longitudinally, perceived PPC has been shown to predict increases in adolescent maladaptive perfectionism over a one-year period (Soenens et al., 2008). Furthermore, Rousseau et al. (2018) attempted to distinguish between dependency-oriented and achievement-oriented psychological control, but found that both dimensions significantly predicted maladaptive perfectionism with minimal differences. In contrast, findings regarding the relationship between PPC and adaptive perfectionism remain inconsistent. Several studies reported non-significant associations (e.g., Soenens et al., 2005, 2008), others found significant positive correlations (e.g., Gong et al., 2016; Smith et al., 2022), and theoretical perspectives suggest PPC might undermine intrinsic striving (Ryan & Reeve, 2024). This inconsistency highlights the need for further investigation into the nature of this relationship.

However, reliance on cross-sectional designs in much of the current research imposes serious limits on predicting this association. First, such designs cannot determine the direction of the effects. It is possible that children with high perfectionism or anxiety traits elicit controlling behaviors from parents rather than the reverse. For instance, parents might become more intrusive and protective in response to the excessive worries displayed by their children (Schittek et al., 2024). Second, when both PPC and perfectionism are reported by adolescents in a single measurement, current emotional states such as depression may lead to a shared method bias where adolescents evaluate both constructs negatively (Van Der Kaap-Deeder et al., 2023). Longitudinal research should be employed to accurately depict how PPC differentially shapes the adaptive versus maladaptive dimensions of perfectionism over time.

## 3. Specific Basic Need Frustration as Mediators

Soenens and Vansteenkiste (2010) regarded PPC as a detrimental parenting environment that globally thwarts adolescents’ basic psychological needs. According to the dual-process model of Self-Determination Theory, need frustration is distinct from the mere lack of need satisfaction. It represents the “dark side” of development—a more toxic experience and a stronger predictor of psychopathology (Kunz & Grych, 2013). Ample evidence has confirmed that PPC actively obstructs basic needs. It undermines relatedness by rendering the parent–child bond conditional (Assor et al., 2004), thwarts autonomy by enforcing compliance through guilt-induction and the invalidation of the child’s perspective, and frustrates competence by imposing unattainable standards that leave adolescents with a chronic sense of inadequacy (Soenens et al., 2005; Soenens & Vansteenkiste, 2010).

Contemporary research has increasingly examined the link between need frustration and perfectionism. Research has identified need frustration as the core mechanism linking evaluative concerns to eating disorders and depression, rather than low satisfaction (Boone et al., 2014; Campbell et al., 2018). Burkitt (2024) demonstrated that need frustration mediates the link between maladaptive perfectionism and psychological impairment. Further, Andrade et al. (2025) confirmed a direct positive correlation between perfectionism and need frustration. However, distinct needs may drive divergent trajectories. Regarding autonomy frustrations, adolescents whose autonomy is thwarted may not rebel, but rather develop learned helplessness or amotivation (Liu et al., 2022). Thus, the internal drive for striving is eroded, leading to a decrease in adaptive personal striving. Conversely, thwarted relatedness and competence are likely to trigger evaluative concerns. Adolescents experiencing this specific frustration tend to base their self-worth on avoiding mistakes and seeking external validation to alleviate relational insecurity and prove their competence (Smith et al., 2022).

Nonetheless, the relationship between need frustration and perfectionism remains debated, particularly regarding the direction of effects. While longitudinal evidence suggests that self-critical perfectionism predicts need frustration (Campbell et al. (2018), theoretical interpretations diverge from the empirical findings; according to the Transdiagnostic Risk Factors framework, need frustration is not merely a pathological outcome but a primary introjected regulation attempt (Ciranka & Van Den Bos, 2021). These attempts often manifest rigid behavioral patterns or personality traits, namely perfectionism. In the context of PPC, we argue that need frustration arises as the psychological cost of controlling parenting, which subsequently triggers perfectionism as a coping response.

Methodologically, existing studies predominantly rely on a single composite score of need frustration, thereby obscuring the potential unique impact of distinct need-frustration subtypes on specific perfectionism dimensions. Moreover, the reliance on cross-sectional designs limits inferences about the direction of effects regarding the role of need frustration in the PPC–perfectionism link. Therefore, it is crucial to examine these specific pathways longitudinally.

## 4. Present Research

To address the ambiguity regarding the longitudinal relationship between PPC and perfectionism and the specific mediating role of need frustration, this study pursued three primary objectives. First, we examined whether PPC longitudinally predicts adolescent perfectionism, specifically aiming to verify how it differentially affects both adaptive and maladaptive facets. Second, we investigated whether need frustration mediates this association, explicitly pinpointing which need-frustration components are operative. Finally, acknowledging that standard longitudinal models often confound stable trait differences with state fluctuations, we employed a random intercept mediation model (RI-Mediation) at the global level to rigorously clarify the general mechanism by disentangling within-person deviation from stable between-person differences.

## 5. Methods

### 5.1. Participants and Procedures

A convenience sampling strategy was employed to recruit participating schools. Three secondary schools consented to participate in the study. Regarding previous research designs (e.g., Soenens et al., 2008; Van Der Kaap-Deeder et al., 2023) and the semester structure in Chinese secondary schools, data collection occurred in three waves at 6-month intervals over one year. After obtaining written informed consent from school administrators, parents, and students, the surveys were administered. Trained psychology graduate students conducted surveys in classrooms, providing standardized instructions and answering questions to ensure data quality. Upon completion, students sealed their own questionnaires in envelopes, which were collected immediately. This study was approved by the Ethics Committee of the Faculty of Psychology, Beijing Normal University and was in accordance with the Declaration of Helsinki (approval No. 201904090037).

The initial assessment at T1 was completed by 1479 adolescents, representing a participation rate of 92.4% among 1600 invited students. Of these, 1454 (98.3% of T1) participated in T2 and 1386 (93.7% of T1) in T3. The final sample consisted of 1432 adolescents, including 663 boys (46%) and 769 girls (54%), who contributed data for at least two of the three waves, with a mean age of 14.60 (*SD_age_* = 1.66). To test systematic bias from sample attrition, we compared participants who completed all three waves with those who missed at least one wave. Independent-sample t-tests and chi-square tests revealed no significant differences between these groups in gender composition (*χ*^2^(1) = 0.73, *p* = 0.39), age (*t* (10.20) = −0.29, *p* = 0.78), or any baseline study variables (all other *p*s > 0.05). These results suggest that attrition was missing at random. Little’s MCAR test was non-significant, supporting the missing-completely-at-random assumption. Accordingly, we used Full Information Maximum Likelihood (FIML) to handle missing data, an approach that utilizes all available information from the sample.

### 5.2. Measures

We utilized validated Chinese versions of established measures where available. For others, a rigorous back-translation protocol was implemented and confirmatory factor analysis was subsequently employed to verify the factorial validity of all newly translated scales.

#### 5.2.1. Parental Psychological Control

Perceived maternal and paternal psychological control was assessed using the Dependency-oriented and Achievement-oriented Psychological Control Scale (Soenens et al., 2010). The scale comprised 17 items distributed across two subscales: dependency-oriented psychological control (DPC; 8 items; e.g., My parents show me that they are disappointed with me if I do not rely on them for a problem) and achievement-oriented psychological control (APC; 9 items; e.g., My parents are less friendly with me if I perform less than perfectly). Items were scored on a 5-point Likert scale ranging from 1 (completely disagree) to 5 (completely agree). In the current investigation, the subscales demonstrated adequate internal consistency at Wave 1, with McDonald’s coefficients of 0.72 and 0.85, respectively. Furthermore, the model yielded a marginal fit to the data, considering the complexity of the item-level structure: *χ*^2^/*df* = 10.37, *p* < 0.001, CFI = 0.87, TLI = 0.84, RMSEA = 0.08, SRMR = 0.06.

#### 5.2.2. Need Frustration

Need frustration was assessed using the 12-item need frustration subscale from Basic Psychological Need Satisfaction and Frustration Scale (Chen et al., 2015), which taps into the frustration of autonomy (4 items, e.g., “Most of the things I do feel like ‘I have to’”), relatedness (4 items, e.g., “I feel excluded from the group I want to belong to”), and competence (4 items, e.g., “I have serious doubts about whether I can do things well”). Responses were made on a 5-point Likert scale (1 = not true at all, 5 = completely true). The Chinese version of this scale has acceptable reliability and validity (Chen et al., 2015). In the present study, McDonald’s *ω* for autonomy, relatedness, and competence frustration at wave 2 was 0.81, 0.75, and 0.71, respectively.

#### 5.2.3. Multidimensional Perfectionism

Perfectionism has been assessed utilizing the Brief Frost Multidimensional Perfectionism Scale (BFMPS; Burgess et al., 2016). This instrument comprises two 4-item subscales: evaluative concerns (e.g., if I fail at work or school, I am a failure as a person) and personal striving (e.g., I set higher goals for myself than most people). Participants rated items on a 5-point Likert scale ranging from 1 (completely disagree) to 5 (completely agree). Within the current sample, both subscales demonstrated acceptable internal consistency, with McDonald’s *ω* of 0.72 and 0.80, respectively. Furthermore, confirmatory factor analysis has yielded a robust model fit: *χ*^2^(12) = 66.03, CFI = 0.97, TLI = 0.95, RMSEA = 0.06, SRMR = 0.04.

#### 5.2.4. Covariates

Data on gender (0 = girl, 1 = boy), age, and socioeconomic status were collected as control variables since these variables might be correlated with the variables of main concern in our study. To measure family socioeconomic status, adolescents were presented with a 10-rung ladder and asked to select where they thought their families were situated based on the average income and occupations of their parents (Adler et al., 2000).

### 5.3. Data Analysis

Data analyses were performed using R (version 4.5.1). Preliminary outlier screening confirmed that all data points fell within the range of ±3 standard deviations (Markatou & Ronchetti, 1997).

Prior to structural testing, measurement model refinement was conducted. We assessed the correlations between parental sources (paternal and maternal) and PPC dimensions (DPC and APC) to determine the appropriate modeling strategy. It revealed an exceptionally high convergence between father- and mother-reported scores for both DPC (*r* = 0.86, *p* < 0.001) and APC (*r* = 0.90, *p* < 0.001), which suggested substantial shared variance. Both DPC and APC total scores exhibited a strong positive correlation (*r* = 0.54, *p* < 0.001). Consistent with these findings, an initial four-indicator model comprising father- and mother-reported DPC and APC yielded poor fit attributable to rater-specific method effects (*χ*^2^ = 2018.83, CFI = 0.68, RMSEA = 0.29). That is, adolescents’ overall response tendencies toward parental figures introduced correlated residuals that obscured substantive distinctions.

Accordingly, aligning with prior research in parenting (Soenens et al., 2005), a parceling strategy was adopted to aggregate father and mother scores into two composite indicators (Total DPC and Total APC). Then, these two indicators were used to define the latent construct of PPC. This approach could capture the cumulative environmental variance while controlling for measurement error, which largely enhanced model parsimony and avoided multicollinearity, particularly given the complexity of the subsequent RI-mediation modeling. Additionally, consistent with the recommendations of Woodfin et al. (2020), the two higher-order dimensions of perfectionism, namely evaluative concerns and personal striving, were modeled as distinct outcomes to allow for the investigation of differential mediation pathways.

For the structural model, nested model comparisons favored the partial mediation model over a restrictive full mediation model (Δ*χ*^2^(2) = 30.21, *p* < 0.001). The final latent variable parallel multiple mediation model therefore examined pathways from T1 PPC to T3 perfectionism via three T2 need frustration mediators. Indirect effects were assessed using bias-corrected bootstrapping with 5000 resamples. To examine generalizability, multi-group analysis tested for gender differences by comparing constrained and unconstrained models. Finally, as a robustness check, an exploratory RI-mediation was estimated to disentangle within-person fluctuations from stable traits.

## 6. Results

### 6.1. Common Method Bias Test

Procedural and statistical controls were implemented to mitigate common method bias. Procedurally, the three-wave time-lagged design temporally separated the measurement of key variables. Statistically, Harman’s single-factor test indicated that the first unrotated factor accounted for only 22.44% of the total variance. These findings suggest that common method bias may not pose a severe threat to present findings (Podsakoff et al., 2003).

### 6.2. Descriptive Statistics and Correlations

Table 1 summarizes the descriptive statistics and bivariate correlations for all variables across T1 to T3. Significant positive correlations were observed between PPC and need frustration dimensions (*r*s = 0.20~0.32, *p*s < 0.01), as did need frustration with evaluative concern (*r*s = 0.23~0.31, *p*s < 0.01). Regarding covariates, subjective socioeconomic status correlated with all study variables, while gender showed negative associations with autonomy frustration, competence frustration, and personal striving.

### 6.3. Testing for Structural Equation Model

**Figure 1:**
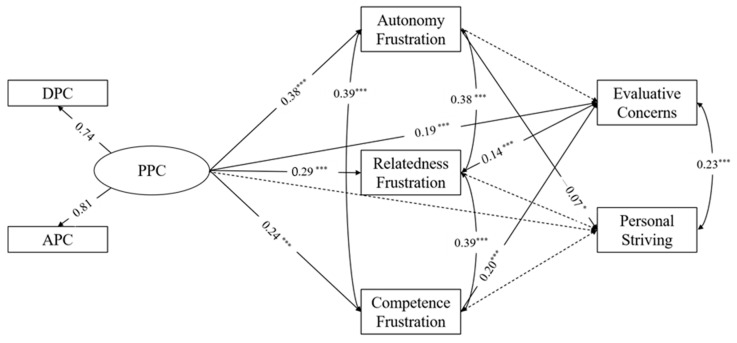
The final latent variable parallel multiple mediation model. Path coefficients are standardized estimates (*β*). Solid arrows indicate significant paths, dashed arrows indicate non-significant paths; *** *p* < 0.001, * *p* < 0.05.

A latent parallel multiple mediation SEM was specified to examine T2 need frustration dimensions (autonomy, relatedness, competence) as mediators between T1 PPC and T3 perfectionism. The model exhibited excellent fit (χ^2^(4) = 9.77, *p* = 0.04; CFI = 0.99; TLI = 0.98; RMSEA = 0.03 [90% CI = 0.01, 0.06]; SRMR = 0.01). Figure 1 details the differential mediation effects.

In the measurement model, both indicators loaded significantly onto the latent PPC variable (DPC = 0.74, APC = 0.81), supporting its construct validity. Structurally, T1 PPC positively predicted all three T2 need frustrations: autonomy (*β* = 0.38), relatedness (*β* = 0.29), and competence (*β* = 0.24; all *p*s < 0.001). Subsequently, T2 relatedness frustration (*β* = 0.14) and competence frustration (*β* = 0.20) predicted T3 evaluative concerns (*p*s < 0.001), whereas autonomy frustration did not (*β* = 0.01; *p* = 0.67). The direct effect from T1 PPC to T3 evaluative concerns remained significant (*β* = 0.19, *p* < 0.001). Conversely, regarding personal striving, only T2 autonomy frustration served as a significant predictor (*β* = −0.07; *p* = 0.04), and neither relatedness nor competence frustration was significant (*p*s > 0.41). The direct effect of T1 PPC on striving was marginally significant (*β* = 0.07, *p* = 0.07).

Bias-corrected bootstrapping with 5000 resamples assessed the indirect effects (Table 2). Significant indirect pathways to evaluative concerns emerged via relatedness frustration (*β* = 0.04, 95% CI [0.01, 0.07]) and competence frustration (*β* = 0.08, 95% CI [0.05, 0.11]); competence frustration accounted for the largest proportion of the total indirect effect (61.5%), followed closely by relatedness frustration (30.8%). For personal striving, significant negative indirect effect was found via autonomy frustration (*β* = −0.03, 95% CI [−0.10, −0.00]). The total indirect effect on evaluative concerns and personal striving was significant separately (*β* = 0.13, 95% CI [0.09, 0.20]; *β* = −0.04, 95% CI [−0.12, −0.02]).

### 6.4. Supplementary Analysis

A supplementary RI-mediation model was estimated using a composite score of need frustration. This approach ensures model parsimony and prevents overparameterization in the between-person covariance matrix. RI-mediation model was estimated to rigorously validate the nature of the longitudinal associations. The model demonstrated excellent fit (*χ*^2^(26) = 35.48, *p* = 0.10; CFI = 0.99; RMSEA = 0.02; SRMR = 0.01). At the within-person level, after controlling for stability, none of the cross-lagged paths from prior PPC to subsequent need frustration, nor from prior need frustration to subsequent perfectionism dimensions, reached statistical significance (all *p*s > 0.10; see Supplementary Table S1). In sharp contrast, the model was robust at the between-person (trait) level. The random intercept of PPC significantly and positively predicted the need frustration random intercept (*β* = 0.62, *p* < 0.001). Need frustration, in turn, predicted higher evaluative concerns (*β* = 0.56, *p* < 0.001) and lower personal striving (*β* = −0.31, *p* < 0.001). Crucially, the trait-level indirect effects were significant for both evaluative concerns (*β* = 0.35, *p* < 0.001) and personal striving (*β* = −0.19, *p* < 0.001).

## 7. Discussion

Utilizing a three-wave longitudinal design spanning one and a half years among Chinese adolescents, the present study constructed a parallel multiple mediation model, complemented by RI-mediation analysis of PPC, psychological need frustration, and perfectionism. Our findings underscored that, even against the backdrop of broad academic pressure and competition, the immediate family environment, specifically PPC, remains a potent predictor of adolescent perfectionism. The empirical results have provided support for the specified frustrated needs framework. It identified basic psychological need frustration as a proximal correlate that has linked controlling parenting environments to stable trait level.

It is noteworthy that PPC is significantly associated with higher evaluative concerns through its links to the frustration of competence and relatedness needs. On the one hand, PPC frequently entails harsh parental evaluation of adolescents’ behaviors and the negation of their perspectives. Such parenting practices directly undermine the need for competence. Precisely, as parents recurrently convey dissatisfaction regarding incorrect behaviors, naive ideas, or they dismiss their ideas, adolescents experience a profound sense of inadequacy. To defend against such inefficacy, they internalize parental standards and develop a rigorous self-monitoring mechanism, manifested as concerns over mistakes (Liu et al., 2022). In this context, perfectionism originates not from a strive for excellence, but a desperate attempt to conceal perceived inadequacy. Adolescents internalize the belief that achieving flawlessness is the only path to restoring competence and appeasing parental criticism. This finding resonates with the work of Soenens et al. (2005), who found that maladaptive perfectionism was identified as being transmitted intergenerationally, with psychological control acting as a pivotal mediator. The current study further specifies such a process; competence frustration represents a core psychological mechanism within this transmission chain, linking chronic controlling environments to internal self-attack (Ryan & Deci, 2017).

Equally critical regarding the mediation of relatedness frustration is that controlling parents often make affection contingent upon children’s obedience or achievement. This practice of parental conditional regard places adolescents in a chronic state of anxiety regarding rejection and abandonment, which directly frustrates their need for relatedness. To maintain the precarious parent–child bond, adolescents adopt the belief that only perfection ensures acceptance (Hewitt & Flett, 1991). Thus, the pursuit of perfection may reflect an attempt to secure relational safety, wherein children seek relational security through flawless performance.

Although the direct effect of parental psychological control on personal striving was marginally significant (*p* = 0.07), the current research demonstrated that parental psychological control negatively predicted personal striving primarily via a distinct autonomy frustration pathway. This finding aligned with the dark pathway of the dual process model within self-determination theory (Vansteenkiste et al., 2020) and was supported by the results of the indirect effect analysis. The core of adaptive perfectionism centers on the pursuit of excellence, which SDT posits must be grounded in autonomous motivation (Vansteenkiste et al., 2010). The present study corroborates this perspective from an inverse standpoint, demonstrating that PPC strongly predicts autonomy frustration. By intruding upon psychological boundaries and imposing parental will, PPC shifts the locus of causality from internal to external (Scharf & Goldner, 2018). Adolescents suffering from autonomy frustration do not merely lack vitality; they may experience amotivation or controlled motivation, losing the intrinsic reason to strive. Further, the cognitive effort required to cope with the guilt and anxiety induced by control substantially depletes psychological resources (Mabbe et al., 2018). Therefore, personal striving is eroded, representing a defensive withdrawal from achievement domains associated with coercion rather than volition.

Methodologically, the application of the RI-mediation model successfully disentangled trait stability from state fluctuations, reinforcing the conceptualization of PPC as a chronic environmental stressor rather than a situational trigger. The finding that mediation effects operate primarily at the between-person level implies that adolescent perfectionism is not driven by transient fluctuations in parental control. Instead, it is profoundly associated with the enduring climate within the family, suggesting that need frustration represents a stable individual-difference factor that links early family environment to long-term personality.

## 8. Implications and Limitations

The present findings could offer critical theoretical and practical implications. First, prior research has predominantly operationalized psychological need satisfaction or frustration as a composite construct. However, the current results demonstrate that distinct types of need frustration precipitate divergent perfectionistic outcomes, underscoring the necessity of examining need-based experiences at a granular level. Such differentiation is critical because specific thwarted needs predict inconsistent developmental trajectories, necessitating precise theoretical mapping.

Practically, these findings challenge the prevailing misconception among parents that psychological pressure serves as a catalyst for achievement. Contrarily, the empirical evidence from this study refutes this notion by establishing a robust pathway from parental psychological control to autonomy frustration and subsequently to diminished personal striving. It is crucial to highlight that coercive tactics not only exacerbate pathological anxiety but also actively erode the very initiative and drive parents seek to cultivate. By frustrating the need for autonomy, such control stifles the internal motivation required for healthy striving. Psychoeducational modules for parent-training programs could highlight the empirical distinction between healthy high standards and psychologically controlling pressure, using concrete examples from the present study’s findings to illustrate the counterproductive effects of conditional regard on adolescent motivation.

Furthermore, these findings suggest that parental psychological control functions as a chronic emotional climate rather than a series of isolated behavioral instances. It is the enduring immersion within a controlling environment, and the resulting sense of thwarted needs, that reflects the development of perfectionistic personality traits. This finding aligns with the emphasis on self-determination theory on the social context as a persistent nurturer or thwarter of development (Ryan & Deci, 2017). Accordingly, merely modifying specific parenting behaviors on a day-to-day basis may prove insufficient if the overarching quality of the parent–child relationship remains unchanged (Van Der Kaap-Deeder et al., 2023). Early and effective interventions require targeting deep-seated, stable patterns of interaction to alleviate the chronic sense of need frustration.

The current research is subject to several limitations. First, regarding methodology, although the study utilizes a longitudinal design, reliance on self-reports limits the objectivity of our data. Future research could employ multi-informant approaches and observer ratings to shed further light on this issue, especially because previous research has shown parents’ and adolescents’ unique and shared perspectives on psychological control (Xu et al., 2024). Then, although the supplementary RI-mediation analysis controlled for autoregressive effects, full autoregressive controls were omitted in the primary analysis. Testing six indirect pathways simultaneously risked severe multicollinearity and model convergence failure. Future studies could strengthen causal inferences by employing autoregressive cross-lagged mediation models. Moreover, while Harman’s single-factor test, the marginal fit of latent PPC, and McDonald’s omega for competence frustration might attenuate observed associations, subsequent investigations could offer more robust evidence regarding the stability of the findings through the application of more rigorous methodological approaches.

Secondly, although RI-mediation results have demonstrated that the mediation process has operated primarily at the between-person level rather than the within-person level, this has not implied that the current findings have merely reflected stable individual differences among adolescents. Because the significant indirect effects have emerged exclusively at the trait level, the observed associations might have reflected stable third influence confounds, especially child effects (Soenens et al., 2008). It has been recognized that adolescents high in perfectionism or presenting specific psychopathology traits might have conversely elicited greater parental controlling behaviors. Although the three wave random intercept mediation design has been superior to cross sectional or two-wave designs, it has not definitively ruled out these possibilities or fully captured these bidirectional microcycles.

Finally, although prior studies recognize the cross-cultural universal impact of PPC, the specific nuances of the Chinese cultural context warrant further scrutiny. In a culture emphasizing filial piety, certain controlling behaviors might be perceived as expressions of parental responsibility; however, the moderation of adolescents’ perception of control remains an open question. This cultural framing may attenuate the negative psychological consequences of PPC, such that Chinese adolescents experiencing comparable levels of parental control to their Western counterparts may exhibit lower levels of need frustration. Conversely, in collectivist cultures where social harmony and family obligation are paramount, the frustration of relatedness may carry even greater effects. Future research should verify the generalizability of this model across diverse cultural contexts.

## 9. Conclusions

The current study employed a three-wave longitudinal design to investigate the differentiated mediating mechanisms of autonomy, relatedness, and competence frustration. The results indicated that need frustration pathways diverged significantly: PPC was positively associated with maladaptive evaluative concerns primarily through competence and relatedness frustration, whereas it was negatively associated with personal striving specifically through thwarted autonomy. Furthermore, the analysis revealed that these associations were driven by stable trait-level factors rather than by temporary state changes. The findings suggest that psychological need frustration is a key mediator between chronic parenting climates and stable perfectionism patterns. The findings also emphasize the necessity of distinguishing between the maladaptive facet (evaluative concerns) and the adaptive facet (personal striving) of perfectionism, as they are predicted by distinct frustrations in basic psychological needs.

## Figures and Tables

**Table 1:** Descriptive statistics and bivariate correlations.

Variables	*M*	*SD*	ICCs	1	2	3	4	5	6	7	8	9
T1 PPC	2.24	0.59	0.59	-								
2. T2 Autonomy Frustration	2.73	0.82	0.49	0.32 ***	-							
3. T2 Relatedness Frustration	2.42	0.74	0.50	0.24 ***	0.45 ***	-						
4. T2 Competence Frustration	3.07	0.77	0.53	0.20 ***	0.44 ***	0.43 ***	-					
5. T3 Evaluative Concerns	2.33	0.70	0.49	0.24 ***	0.23 ***	0.28 ***	0.31 ***	-				
6. T3 Personal Striving	2.76	0.73	0.58	0.03	−0.06 *	−0.03	−0.04	0.23 ***	-			
*Covariates*												
7. T1 Gender	-	-		−0.03	−0.07 *	−0.01	0.13 ***	0.00	−0.08 **	-		
8. T1 Age	14.60	1.66		0.04	0.07 **	0.00	0.11 ***	0.01	0.01	0.03	-	
9. T1 SES	5.51	1.34		−0.11 ***	−0.14 ***	−0.14 ***	−0.16 ***	−0.07 **	0.07 **	−0.01	−0.22 ***	-

***Note.*** * *p* < 0.05, ** *p* < 0.01, *** *p* < 0.05, T1~T3 means T1 to T3, SES means family socioeconomic status, ICCs means intraclass correlation coefficients.

**Table 2:** Standardized coefficients and 95% confidence intervals for indirect effects.

Pathways	Std. Estimate	95% Confidence Interval (CI)	
		Lower	Upper
*Indirect pathway to evaluative concerns*			
PPC(T1) → Autonomy Frustration(T2) → Evaluative Concerns(T3)	0.01	−0.03	0.05
PPC(T1) → Relatedness Frustration(T2) → Evaluative Concerns(T3)	0.04 ^a^	0.01	0.07
PPC(T1) → Competence Frustration(T2) → Evaluative Concerns(T3)	0.08 ^a^	0.05	0.11
Total indirect effects on Evaluative Concerns	0.13 ^a^	0.09	0.20
*Indirect pathway to personal striving*			
PPC(T1) → Autonomy Frustration(T2) → Personal Striving(T3)	−0.03 ^a^	−0.10	−0.00
PPC(T1) → Relatedness Frustration(T2) → Personal Striving(T3)	0.00	−0.04	0.03
PPC(T1) → Competence Frustration(T2) → Personal Striving(T3)	−0.01	−0.04	0.02
Total indirect effects on Personal Striving	−0.04 ^a^	−0.12	−0.02

***Note.* **^a^ represents 95% CI does not include zero; T1~T3 means T1 to T3.

## Data Availability

The data presented in this study are available on request from the corresponding author for scientific purposes.

## Supplementary Materials

The following supporting information can be downloaded at: https://www.mdpi.com/article/10.3390/bs16071145/s1, Table S1: Standardized Estimates and 95% Confidence Intervals for the Random Intercept Mediation Model.
