# Supplementary material for: Divergent Mechanisms Linking Parental Psychological Control to Perfectionism Among Adolescents: The Longitudinal Role of Need Frustration
Source: Behav Sci (Basel). 2026 Jul 8;16(7):1145. doi: 10.3390/bs16071145 (PMC13403983; doi:10.3390/bs16071145)
Supplement: Supplementary file 1 [file behavsci-16-01145-s001.zip › behavsci-4349958-supplementary.pdf]

Supplementary Table S1.

Standardized Estimates and 95% Confidence Intervals for the Random Intercept Mediation Model

Pathways	Within-Person Std. Estimate	95% CI	Between-Person Std. Estimate	95% CI
Predictor to Mediator				
PPC→Need Frustration	.05	[-.05, .15]	$.62^{***}$	[.52, .65]
Mediator to Outcomes				
Need Frustration→Evaluative Concerns	.06	[-.03, .15]	$.56^{***}$	[.44, .58]
Need Frustration→Personal Striving	-.04	[-.13, .05]	$-.31^{***}$	[-.35, -.28]
Direct Effects				
PPC→Evaluative Concerns	.06	[-.03, .14]	$.24^{***}$	[.12, .26]
PPC→Personal Striving	.02	[-.04, .08]	$.11^{*}$	[.11, .19]
Indirect Effects				
PPC→Need Frustration→Evaluative Concerns	.00	[-.02, .03]	$.35^{***}$	[.24, .37]
PPC→Need Frustration→Personal Striving	.00	[-.03, .04]	$-.19^{***}$	[-.23, -.11]

Note. $^{*}p<0.05$, $^{***}p<.001$.
